# Inter-American Society of Cardiology (CIFACAH-ELECTROSIAC) and Latin-American Heart Rhythm Society (LAHRS): multidisciplinary review on the appropriate use of implantable cardiodefibrillator in heart failure with reduced ejection fraction

**DOI:** 10.1007/s10840-022-01425-4

**Published:** 2022-12-05

**Authors:** Juan Esteban Gómez-Mesa, Manlio Márquez-Murillo, Marcio Figueiredo, Ana Berni, Ana Margarita Jerez, Elaine Núñez-Ayala, Freddy Pow-Chon, Luis Carlos Sáenz-Morales, Luis Fernando Pava-Molano, María Claudia Montes, Raúl Garillo, Stephania Galindo-Coral, Walter Reyes-Caorsi, Mario Speranza, Alexander Romero

**Affiliations:** 1 Cardiology Department, Fundación Valle del Lili, Cali, Colombia; 2 Centro de Investigaciones Clínicas, Fundación Valle del Lili, Cali, Colombia; 3 Inter-American Council of Heart Failure and Pulmonary Hypertension/CIFACAH, Mexico City, Mexico; 4 Inter-American Society of Cardiology/IASC, Mexico City, Mexico; 5 Instituto Nacional de Cardiología Ignacio Chávez, Mexico City, Mexico; 6 Inter-American Council of Electrocardiography and Arrhythmias/ELECTROSIAC, Mexico City, Mexico; 7 University of Campinas (UNICAMP) Hospital, Campinas, Brazil; 8 Latin American Heart Rhythm Society/LAHRS, Montevideo, Uruguay; 9 Hospital Angeles Pedregal, Mexico City, Mexico; 10 Instituto de Cardiología Y Cirugía Cardiovascular, La Habana, Cuba; 11 Electrophysiology, Arrhythmias and Pacemaker Unit, CEDIMAT, Centro Cardiovascular, Santo Domingo, Dominican Republic; 12 Hospital Luis Vernaza, Guayaquil, Ecuador; 13 Fundación Cardioinfantil–Instituto de Cardiología, Bogotá, Colombia; 14 Facultad de Ciencias Médicas, Pontificia Universidad Católica Argentina, Buenos Aires, Argentina; 15 Comisión Honoraria Para La Salud Cardiovascular, Montevideo, Uruguay; 16 Hospital Clínica Bíblica, Ciudad de Costa Rica, Costa Rica; 17 Hospital Santo Tomas, Ciudad de Panama, Panama

**Keywords:** Arrhythmia, Chronic kidney diseases, Heart failure, Reduced ejection fraction, Implantable cardioverter defibrillator, Sudden cardiac death

## Abstract

**Background:**

Our main objective was to present a multidisciplinary review on the epidemiology of sudden cardiac death (SCD) and the tools that could be used to identify malignant ventricular arrhythmias (VAs) and to perform risk stratification. In addition, indications and contraindications for the use of implantable cardioverter defibrillator (ICD) in general and in special populations including the elderly and patients with chronic kidney disease (CKD) are also given.

**Methods:**

An expert group from the Inter American Society of Cardiology (IASC), through their HF Council (CIFACAH) and Electrocardiology Council (ElectroSIAC), together with the Latin American Heart Rhythm Society (LAHRS), reviewed and discussed the literature regarding the appropriate use of an ICD in people with heart failure (HF) with reduced ejection fraction (HFpEF). Indications and contraindications for the use of ICD are presented in this multidisciplinary review.

**Results:**

Numerous clinical studies have demonstrated the usefulness of ICD in both primary and secondary prevention of SCD in HFpEF. There are currently precise indications and contraindications for the use of these devices.

**Conclusions:**

In some Latin American countries, a low rate of implantation is correlated with low incomes, but this is not the case for all Latin America. Determinants of the low rates of ICD implantation in many Latin American countries are still a matter of research. VA remains one of the most common causes of cardiovascular death associated with HFrEF and different tools are available for stratifying the risk of SCD in this population.

**Supplementary Information:**

The online version contains supplementary material available at 10.1007/s10840-022-01425-4.

## Introduction

Heart failure (HF) is observed with increasing frequency in the American continent, and sudden cardiac death (SCD) remains the most common form of cardiovascular death in HF with reduced ejection fraction (HFrEF). Despite improvements in medical treatment, the use of an implantable cardioverter defibrillator (ICD) is still necessary to prevent arrhythmic SCD. Therefore, the Inter American Society of Cardiology (IASC), through their HF Council (CIFACAH) and Electrocardiology Council (ElectroSIAC), together with the Latin American Heart Rhythm Society (LAHRS) has supported a Multidisciplinary Review on the appropriate use of these devices in this group of patients.

The relevance of the topic is based on the fact that an efficient use of resources must be implemented in all countries of the American continent. In some countries, their use seems to be excessive, while in other regions, its use is practically null; these differences may be related to the variable economic income of the population and may be also related to a “medical component” (infrequent recommendation) that could explain the low rate of ICD implants in some Latin Americans countries, possible due to lack of discussion by physicians as an available treatment option in eligible patients. Therefore, IASC and LAHRS have considered essential, as continental societies, to undertake this Multidisciplinary Review that can serve as a guide and orientation for all those doctors involved with the care of patients with HF. In the present work, a summary of epidemiology of SCD, a review of the tools that could be used to identify malignant ventricular arrhythmias (VA) and to perform risk stratification is presented. In addition, indications and contraindications for the use of ICD in general and in special populations, including the elderly and patients with chronic kidney disease (CKD), are also presented. The manuscript was not subjected to full external peer review and represents the own view of the society/work group.

## Epidemiology of sudden death in heart failure

Sudden death (SD) is one of the most common causes of cardiovascular events (up to 70–80%) in patients with HFrEF and less frequent (up to 30%) in patients with HF with preserved ejection fraction (HFpEF) [1].

Due to the lack of a universal definition of SD, its incidence and prevalence is variable in different clinical studies and registries. On the other hand, the accepted definition of SCD is death that occurs within one hour after the onset of symptoms in witnessed cases or within 24 h after the last time the person was seen alive when there are no witnesses [2]. Most deaths are not witnessed, with ventricular fibrillation (VF) and asystole being the final underlying mechanism [3] and constituting the reason behind considered “shockable “or “not-shockable” rhythms in the new algorithms of cardiac arrest. Worldwide, sudden and unexpected cardiac death is the most common cause of death, accounting for 17 million deaths each year, with SCD accounting for 25% of these. An estimated 180,000 to 300,000 episodes of SCD occur annually just in the USA.

Despite the decrease in total cardiovascular deaths in the recent decades [4], due to better preventive strategies, the incidence of SCD as a cause of cardiovascular death has increased. This has occurred in part because hospital mortality has decreased overall, highlighting the need to improve risk stratification methods and preventive strategies.

Economic deprivation is considered as an independent risk factor for HF [5, 6]. In Latin America (LA), we can find countries with a wide range of socioeconomic development and there are specific risk factors for HF with a higher incidence in this region, such as hypertension, rheumatic fever, and Chagas disease. The prevalence of HF in LA is estimated at 1.0–2-0% and in Europe and the USA it ranges between 1.0 and 14% [7–9]. A retrospective cohort study from Mexico City reported an in‐hospital mortality of 17.9% in patients with acute HF and all‐cause in‐hospital mortality being higher among patients with acute HF (AHF) compared with patients without AHF (17.9% vs. 5.0%; *P* < 0.0001) [10].

Chagas disease is an endemic disease in LA caused by the *Trypanosoma cruzi*. Almost 18 million people are infected, ∼25% of them developing chronic myocardial disease after years or decades. The main causes of death in this population are congestive HF and SCD due to dilated cardiomyopathy. Malignant ventricular arrhythmias are thought to be the main cause of SCD, bradyarrhythmias and thrombo-embolic events also account for some of this SCD. However, the efficacy and safety of these devices in patients with Chagas disease have been poorly studied. Eighty-nine chagasic patients with ICD were included for analysis from the Medtronic ICD Registry LA. 91% had secondary prevention indications. Mean left ventricular ejection fraction was 40 ± 11%; during follow-up, 6.7% died (6.7%); one due to SCD. Forty-two percent received appropriate ICD therapies. A total of 737 episodes were detected by the ICD. The mean period between ICD implantation and the first appropriate therapy was 104 days. Electrical storms were observed in 7%. Inappropriate therapies were observed in seven patients [11].

## Pathophysiology of sudden death in heart failure

In SCD, the abrupt collapse of the circulation is often attributed to sustained ventricular tachycardia (VT) or VF [12]. Patients with ischemic HF and ventricular dilatation who have coronary artery disease (CAD) develop myocardial scarring that acts as a structural arrhythmogenic substrate, allowing a macro-reentry mechanism at the border between the normal myocardium, the scar, and the fibrotic area. Ventricular arrhythmias (VAs) occur as a result of functional areas of slow conduction of ventricular depolarization wavefronts in scar territories [13]. On the other hand, HF patients without a history of CAD present with progressive cardiac remodeling that causes an increased myocardial tension, leading to small compensatory mechanisms that maintain synchronized contraction. Interdependence maintains electrical stability until the occurrence of the weakening of the focal mechanism that exerts pressure and stabilizes the ability of cardiomyocytes to achieve synchronized contraction, resulting in acute failure of the cardiac mechanical function [14]. Finally, VA can occur without an identifiable trigger, be precipitated by a sudden increase in catecholamines levels, hyperactivity of the sympathetic nervous system, fluid and electrolyte imbalance, or by the use of medications with proarrhythmic effects [15]. In a small subset of patients, VA can occur without an identifiable trigger. Regardless of the cause, these VA can be reverted by appropriate therapy from an ICD. However, ICD detection algorithms and therapies are not infallible and not all SCD is preventable. In randomized clinical trials, candidates for ICD implantation experienced a variable reduction in the risk of SCD, 50% in patients with non-ischemic cardiomyopathy and 60–70% in patients with ischemic heart disease (IHD) [16]. In 30–50% of patients with sudden circulatory collapse, findings on the electrocardiogram (ECG) included bradyarrhythmia, asystole, or electromechanical dissociation; also, events attributed to acute HF could be found, especially in patients with severely remodeled hearts. These episodes cannot always be prevented with an ICD [17].

## Diagnosis of ventricular arrhythmias (how to perform an investigation of a survivor of a cardiac arrest)

VAs are a common cause of exacerbation of symptoms in HF, as well as discharges (appropriate and inappropriate) in patients with ICD. Diagnostic methods for rhythm disorders are useful to correlate symptoms (syncope / near syncope) with VA, stratify risk of SCD and, in particular cases, evaluate the response to antiarrhythmic treatment [18].

Diagnostic methods recommended in clinical practice:

- 12-lead ECG at rest: it is the initial routine study to evaluate electrical disorders in structural heart disease, with findings such as dilatation of cavities, presence of Q waves (old infarction), elevation / depression of the ST segment (acute ischemia), bundle branch block, QT interval alterations and other repolarization disorders. Fragmented QRS is a specific marker of myocardial scar and high risk of SD due to VA (VT / VF) and appropriate discharge of the ICD, both in IHD and in non-ischemic dilated cardiomyopathy (DCM) [19, 20]. The presence of epsilon waves (V1–V3), QRS duration > 110 ms, and inverted T waves in V2–V3 suggest arrhythmogenic right ventricular cardiomyopathy (ARVC).
- The ECG during tachycardia: in case of hemodynamic stability, it allows to assess the morphology of the tachycardia (monomorphic / polymorphic), to estimate the location of the arrhythmogenic circuit, and to evaluate the adequate programming of the ICD according to the cycle length of the clinical tachycardia. Electrocardiographic criteria that support the diagnosis of VT include: AV dissociation, duration of the QRS complex greater than 140 ms, monophasic R wave in aVR, morphological criteria of the QRS complex such as positive or negative concordance in precordial, absence of RS waves in precordial leads, RS interval > 100 ms in at least one precordial lead, among others [21–23].
- Stress test: it is useful in patients with symptoms triggered by activity, as well as to evaluate the behavior of arrhythmias during exercise. Exertional-triggered arrhythmias such as catecholaminergic polymorphic VT (CPVT) generally present in the absence of structural heart disease [24].
- Signal averaged electrocardiogram: it is most useful in ARVC, since it identifies late ventricular potentials originating in the scar area; a positive test is part of the diagnostic criteria for the disease. In the case of IHD, the routine use of this test is not recommended, since the positive predictive value is low for SD risk stratification [25, 26].
- Holter-electrocardiographic-monitoring: useful in patients with symptoms occurring at least once a day. This diagnostic method allows a better analysis of VA: premature ventricular complexes as triggers of sustained arrhythmias, arrhythmic load (% of extrasystoles and / or VT in 24 h), morphology of clinical tachycardia and relationship of VA with deterioration of systolic function. Non-sustained VT (NSVT) can be found in up to 60–80% of patients with HFrEF. In the acute phase of myocardial infarction (MI), NSVT has not been shown to be an independent factor for poor prognosis; however, its presence after 48 h after the acute event is associated with increased morbidity and mortality. In the case of HF with a moderately decreased EF (35–40%), NSVT may indicate disease progression [27–29]. In addition to VA, it is common to find atrial fibrillation (AF) during this monitoring; its prevalence is 25–37% in outpatient patients with HF and up to 20% of hospitalized patients may have a first episode of AF [12, 30]. Detection of AF is relevant, since it can be the cause of inappropriate discharge in patients with ICD. Besides that, thromboembolic risk stratification and the use of oral anticoagulants should be carried out in accordance with international clinical practice guidelines [31, 32].
- Implantable monitoring devices: provide up to 25% more diagnostic sensitivity in continuous monitoring of outpatients whose symptoms are rare, and the results of conventional methods are inconclusive. In patients with syncope, it allows to associate the symptoms with VA. In the CARISMA study, continuous monitoring in patients with EF < 40% with a 2-year follow-up after MI, demonstrated arrhythmias in up to 46% of the subjects, most of them asymptomatic; however, routine use is not recommended for monitoring asymptomatic VA [33]. On the other hand, it is a very useful diagnostic method for the detection of AF in patients with HF.
- T-wave microvoltage alternans: it is the oscillation in voltage change of the T wave from beat to beat, which is an expression of the heterogeneity of repolarization and risk of arrhythmias. As an isolated parameter for SD risk stratification, it has limited prognostic value, however, associated with other markers (heart rate turbulence, EF, electrophysiological study) it can be a good predictor of SD and appropriate shocks in specific populations, such as IHD [34].
- Electrophysiological study: this invasive test allows risk stratification in SD by inducing VT / VF with programmed ventricular pacing protocols. In the MUST study, in patients with IHD, EF < 40% and NSVT an electrophysiological study was performed to select ICD therapy in those with sustained VT induction. The analysis showed a high positive predictive value for the electrophysiological study, with a low negative predictive value [35]. The ABCD study, in patients with IHD, EF < 40% and NSVT, demonstrated that the combination of a negative electrophysiological study with negative T-wave alternation identifies a low-risk population with a 2.3% risk / event rate at 2 years [36]. Currently, the electrophysiological study is recommended as a complementary test in the selection of candidates for ICD in a certain group of patients.

Finally, in patients with cardiac pacing devices, it is possible to monitor VA or AF according to the programmed detection zones; remote monitoring of high-energy devices facilitates early identification of clinically significant arrhythmias [37].

## Implantable cardioverter defibrillator: clinical trials confirms the benefit

Medical therapy with class Ic antiarrhythmic agents or amiodarone to prevent SCD can be ineffective in some cases [38, 39]. Because of that, the most significant advance in the prevention of SCD has been the development of ICD [40]. Secondary prevention clinical trials comparing antiarrhythmic drugs vs ICD [41–43] have shown statistically significant improved survival rates with ICD implantation compared with drug treatment.

The Multicenter Automatic Defibrillator Implantation Trial I (MADIT I) [44] and the Multicenter Unsustained Tachycardia Trial (MUSTT) [35] enrolled patients after MI and compared primary prevention with an ICD vs standard medical treatment in patients with HFrEF (< 35% and < 40%, respectively) plus documented or induced VT. They demonstrated a 58 to 59% relative risk (RR) reduction of death. Subsequently, the MADIT II trial [45] showed a 28% RR reduction in mortality at 2 years in post-MI patients with EF < 30% without the requirement of documented or induced VT (Tables 1 and 2).

**Table 1:** Principal trials of ICD in heart failure: baseline characteristics

Trial	Acronyms	Year	# Patients	Patients included	LVEF (%)	Follow-up time (months)
Primary prevention						
Multicenter Automatic Defibrillator Implantation Trial [44]	MADIT I	1996	196	Patients with NYHA FC I, II or III with prior MI	< 35	27
GABG-Patch Trial [47]	GABG-PATCH	1997	900	Elective CABG in patients with < 80 years	≤ 35	36
Multicenter Unsustained Tachycardia Trial [35]	MUSTT	1999	704	Patients with coronary artery disease	≤ 40	39
Multicenter Automatic Defibrillator Implantation Trial [45]	MADIT II	2002	1232	Patients > 21 years old with a prior MI and NYHA FC I, II or III	< 30	20
The Defibrillator in Acute Myocardial Infarction Trial [104]	DINAMIT	2004	674	Patients between 18–80 years old and a recent MI (6–40 days previously) and impaired cardiac autonomic function (manifested as depressed heart-rate variability or and elevated average 24-h heart rate on Holter monitoring)	< 35	30
Sudden Cardiac Death in Heart Failure Trial [38]	SCD-HeFT	2005	2521	Patients with ischemic or non-ischemic HF, NYHA FC II or III in CMT	≤ 35	45.5
Secondary prevention						
The Antiarrhythmics versus Implantable Defibrillators Investigators [41]	AVID	1997	1016	Patients who had been resuscitated from near-fatal VF or who had undergone cardioversion from sustained VT. Patients with VT also had either syncope or other serious cardiac symptoms	≤ 40	18
The Cardiac Arrest Study Hamburg [43]	CASH	2000	288	Patients resuscitated from cardiac arrest secondary to documented sustained ventricular arrhythmias	46	57
Canadian Implantable Defibrillator Study [42]	CIDS	2000	659	Patients with resuscitated VF, VT or with monitored syncope	≤ 35	36
Non-ischemic dilated cardiomyopathy						
Cardiomyopathy Trial [49]	CAT	2002	104	Patients between 18 and 70 years old, recent onset of DCM (≤ 9 months) and NYHA FC II or III	< 30	24
The Defibrillators in Non-Ischemic Cardiomyopathy Treatment Evaluation [46]	DEFINITE	2004	458	Patients with ambient arrhythmias, a history of symptomatic HF, and the presence of non-ischemic DCM	< 36	29
Defibrillator Implantation in Patients with Non-Ischemic Systolic Heart Failure [16]	DANISH	2016	1116	Patients with symptomatic systolic HF (LVEF ≤ 35%) not caused by coronary artery disease (58% received cardiac resynchronization therapy)	≤ 35	67.6

*CABG* coronary artery bypass graft; *CMT* conventional medical therapy; *DCM* dilated cardiomyopathy; *FC* functional class; *HF* heart failure; *ICD* implantable cardiodefibrillator; *LVEF* left ventricular ejection fraction; *MI* myocardial infarction; *NYHA* New York Heart Association; *VF* ventricular fibrillation; *VT* ventricular tachycardia

**Table 2:** Principal trials of ICD in heart failure: outcomes

Trial	Acronyms	Electrocardiographic findings	Interventions	Conclusions
Primary prevention				
Multicenter Automatic Defibrillator Implantation Trial [44]	MADIT I	Documented asymptomatic NSVT	ICD vs CMT	ICD was associated with a mortality reduction of 54%; In the subgroup of patients with LVEF 26%-35%, there were no significant improvement in mortality with DAI
GABG-Patch Trial [47]	GABG-PATCH	Abnormalities on a signal-averaged ECG	ICD + CABG vs CABG alone	No improvement in survival with placement of ICD
Multicenter Unsustained Tachycardia Trial [35]	MUSTT	Sustained, monomorphic VT induced by any method of stimulation	Antiarrhythmic therapy (including drugs) vs ICD	Antiarrhythmic therapy with ICD, but not with antiarrhythmic drugs, reduces the risk of SC or death (27%) in high-risk patients with coronary disease
Multicenter Automatic Defibrillator Implantation Trial [45]	MADIT II	Frequent or repetitive ventricular ectopic beats during 24-h Holter monitoring (eliminated criteria)	ICD vs CMT	Prophylactic implantation of a ICD improves survival (31%)
The Defibrillator in Acute Myocardial Infarction Trial [99]	DINAMIT	Standard deviation of normal-to-normal RR intervals of < 70 ms or a mean RR interval of < 750 ms	ICD vs conventional medical treatment	Prophylactic ICD therapy does not reduce overall mortality in high-risk patients who have recently had a MI. ICD therapy was associated with a reduction in the rate of death due to arrhythmia
Sudden Cardiac Death in Heart Failure Trial [38]	SCD-HeFT	All patients underwent electrocardiography and 24-h ambulatory ECG	CMT / Placebo vs CMT / Amiodarone vs CMT / ICD	Amiodarone has no favorable effect on survival, whereas single-lead, shock-only ICD therapy reduced overall mortality by 23%
Secondary prevention				
The Antiarrhythmics versus Implantable Defibrillators Investigators [41]	AVID	VF or sustained VT	ICD vs class III antiarrhythmic drugs (primarily amiodarone)	ICD is superior to antiarrhythmic drugs for increasing overall survival
The Cardiac Arrest Study Hamburg [43]	CASH	VF or VT	ICD vs antiarrhythmic drugs (Amiodarone or Propafenone or Metoprolol)	Therapy with an ICD is associated with a 23% (non-significant) reduction of all-cause mortality rates when compared with treatment with amiodarone/metoprolol
Canadian Implantable Defibrillator Study [42]	CIDS	VF or VT	ICD or Amiodarone	A 20% relative risk reduction occurred in all-cause mortality and a 33% reduction occurred in antiarrhythmic mortality with ICD therapy compared with amiodarone; this reduction did not reach statistical significance
Non-ischemic dilated cardiomyopathy				
Cardiomyopathy Trial [49]	CAT	Exclusion criteria: History of symptomatic bradycardia, VT or VF	ICD vs control	Did not provide evidence in favor of prophylactic ICD implantation in patients with DCM of recent onset and impaired LVEF
The Defibrillators in Non-Ischemic Cardiomyopathy Treatment Evaluation [46]	DEFINITE	Ambient arrhythmias: Episode of non-sustained VT on Holter monitoring (3 to 15 beats at a rate > 120 bpm) or an average of at least 10 premature ventricular complexes per hour	ICD vs CMT	Implantation of a ICD significantly reduced the risk of sudden death from arrhythmia and was associated with a non-significant reduction in the risk of death from any cause
Defibrillator Implantation in Patients with Non-Ischemic Systolic Heart Failure [16]	DANISH	Exclusion criteria: Permanent atrial fibrillation with a resting heart rate higher than 100 bpm	ICD vs CMT	ICD was not associated with a significantly lower long-term rate of death from any cause than was usual clinical care

*CABG* coronary artery bypass graft; *CMT* conventional medical therapy; *ECG* electrocardiography; *ICD* implantable cardiodefibrillator; *LVEF* left ventricular ejection fraction; *NSVT* non-sustained ventricular tachycardia; *VF* ventricular fibrillation; *VT* ventricular tachycardia

The DEFINITE trial (Defibrillators in Non-Ischemic Cardiomyopathy Treatment Evaluation) [46] compared the benefit of ICD vs standard therapy in patients with HF (EF ≤ 35%) and premature ventricular contractions or NSVT, showing a strong trend toward reducing mortality with ICD. Also, the SCD-HEFT trial (Sudden cardiac death in heart failure) [38] included both ischemic and non-ischemic cardiomyopathy patients with NYHA (New York Heart Association) functional class (FC) II–III and EF ≤ 35%, and reported a benefit from ICD compared to standard medical treatment (Tables 1 and 2) [47–49].

A crucial aspect in primary prevention studies with ICD is that besides low EF, no other significant risk predictor identifies patients who may benefit from ICD implantation.

Using these studies as a guide to prescribe an ICD means that we have only targeted a subgroup of patients where the incidence of events is high, and therefore, they have been labeled as high risk. Most SCD episodes occur in individuals who, before the event, have not known heart disease and are not known to be high-risk patients by traditional measures or present as a manifestation of an underlying heart condition undiagnosed. That means that, in the general population, the majority of SCD events occur in patients considered “low risk” for events [50]. Although the incidence within this group of patients is low, they represent the largest number of events cumulatively. Indications for primary prevention in infrequent conditions such as hypertrophic cardiomyopathy (HCM), ARVC, long QT syndrome (LQTS), Brugada syndrome, and early repolarization remain less clear. They do not have definitive definitions and defined risk markers beyond the patient's symptoms [51].

In LA, international guidelines for primary prevention ICD implantation are not well followed. The main reason is that cardiologists believe that patients do not meet indication criteria, even though the PLASMA (Probabilidad de Sufrir Muerte Arrítmica) study data confirm that criteria are met [52].

The ICD-LABOR study reported the experience of seven LA countries and 770 patients in the secondary prevention of SCD; every patient presented with antecedents of aborted SCD or cardiac arrest due to VT or VF. Patients included fulfilled the Class I indication for ICD. Despite the differences in terms of pathologies between the ICD-LABOR and randomized ICD trials, a parallel evolution in all-cause mortality and cardiac mortality was observed. Independent risk factors for mortality included age > 70 years, male gender, NYHA III/IV, and ejection fraction < 0.30. The etiology of heart disease (Chagas vs Coronary Disease) was not found to be a risk factor [53].

## Indications and contraindications for implantable cardioverter defibrillator

Deaths from cardiac diseases have been diminishing in the industrialized world during the last two decades. People live longer and comorbidities as IHD or DCM are growing, and they are associated with SCD. It is estimated that about 20% of all deaths still occur suddenly and unexpectedly, most often caused by VF or asystole [4]. ICD is reliable aborting SCD due to VF, and intravascular ICDs have the ability to terminate reentrant monomorphic VTs using anti-tachycardia pacing. ICDs have been tested in randomized studies in populations with ischemic/not ischemic cardiomyopathies or SCD survivors [54]. For selected patients, ICD may have cardiac resynchronization therapy (CRT) pacing, improving the health condition of the patient. There are no direct comparison trials between ICD and CRT [55].

The ICD is recommended in two scenarios: 1) *secondary prevention*: when the patient has had a major arrhythmic event as cardiac arrest due to VF or hemodynamically unstable VT; 2) *primary prevention*: refers to patients with risk of dying of major arrhythmic events but with no documented previous major arrhythmias.

### Problems in the ICD selection

Randomized studies included patients with or without ischemic cardiomyopathy, but patients with other cardiomyopathies (e.g., HCM or ARVC) or channelopathies (e.g., Brugada or LQTS) are not represented in randomized studies; in these cases, the decision of implanting an ICD is based on recommendations of experts. Most instances of SCD actually occur in the general population and patients diagnosed with markers of SCD are at higher risk [56].

There are several markers indicating risk of SCD, and the most remarkable are low EF, NYHA FC, elevated heart rate, frequent premature ventricular complexes, NSVT, myocardial aneurysm, extensive scarring, and prolonged QRS duration on the ECG. Indicators of autonomic dysfunction and electrical alterations are diminished heart rate variability, abnormal baroreflex sensitivity, heart rate turbulence, microvolt T-wave alternans and neurohormonal markers as B-type natriuretic peptide. Except left ventricular (LV) EF and NYHA FC, others markers are not generally used because of their lack of predictive value and lack of robust evidence [15].

### Indications and contraindications

For primary prevention indications, current guidelines emphasize the importance of receiving optimal medical therapy (OMT) in HF patients generally for at least three months prior to considering ICD device implantation in eligible patients based on LVEF criteria alone and at least > 40 days post-MI. Patient should also have a life expectancy > 1 year with good NYHA FC and quality-of-life issues should be discussed before implantation. Other aspects to consider before ICD implantation are dual versus single coil ICD, ICD versus CRT-D, and endocardial or entirely subcutaneous.

Recommendations for implanting ICD [20, 57, 93] are shown in Tables 3 and 4. A Spanish version of this recommendations is presented in the Supplemental Material 1.

**Table 3:** Recommendations for implanting ICD

Class of recommendation	Recommendation
Primary prevention	
IA	An ICD is recommended to reduce the risk of sudden death and all-cause mortality in patients with symptomatic HF (NYHA class II III) of an ischemic aetiology (unless they have had a MI in the prior 40 days—see below), and an LVEF < _35% despite > _3 months of OMT, provided they are expected to survive substantially longer than 1 year with good functional status
IIa A	An ICD should be considered to reduce the risk of sudden death and all-cause mortality in patients with symptomatic HF (NYHA class II III) of a non-ischemic aetiology, and an LVEF < _35% despite > _3 months of OMT, provided they are expected to survive substantially longer than 1 year with good functional status
IIa A	Patients should be carefully evaluated by an experienced cardiologist before generator replacement, because management goals, the patient’s needs and clinical status may have changed
IIb B	A wearable ICD may be considered for patients with HF who are at risk of sudden cardiac death for a limited period or as a bridge to an implanted device
III A	ICD implantation is not recommended within 40 days of a MI as implantation at this time does not improve prognosis
III C	ICD therapy is not recommended in patients in NYHA class IV with severe symptoms refractory to pharmacological therapy unless they are candidates for CRT, a VAD, or cardiac transplantation
Secondary prevention	
IA	An ICD is recommended to reduce the risk of sudden death and all-cause mortality in patients who have recovered from a ventricular arrhythmia causing hemodynamic instability, and who are expected to survive for > 1 year with good functional status, in the absence of reversible causes or unless the ventricular arrhythmia has occurred < 48 h after a MI

**Table 4:** Primary prevention ICD implant indications at pediatric age

		Recommendations
General Recommendation	IIb	Patients with genetic cardiovascular diseases and risk factors for SCA or pathogenic mutations and family history of recurrent SCA
Long QT	IIb	Patients with established clinical risk factors and/or pathogenic mutations
CPVT	IIb	Polymorphic/bidirectional VT despite optimal pharmacological therapy with or without cardiac sympathetic denervation
BrS	IIa	Spontaneous type I Brugada ECG pattern and recent syncope presumed due to ventricular arrhythmias
	IIb	Syncope presumed to be due to ventricular arrhythmias and type I Brugada pattern ECG only with provocative medications
HCM	IIa	Children with ≥ 1 primary risk factors, syncope, massive left ventricular hypertrophy, non-sustained VT, or family history of early HCM-related SCD and after considering potential complications of long-term ICD placement
	IIb	Without the above risk factors but with secondary risk factors for SCA such extensive LGE on cardiac MRI or systolic dysfunction
NIDCM	IIb	Syncope or an LVEF ≤ 35% despite optimal medical therapy
CHD	IIb	Unexplained syncope in the presence of ventricular dysfunction, non-sustained VT, or inducible arrhythmias at electrophysiological study
	IIb	Patients with single or systemic right ventricular ejection fraction < 35% particularly in the presence of additional risk factors such as VT, arrhythmic syncope, or severe systemic AV valve insufficiency
ACM	IIb	Inherited ACM associated with increased risk of SCD based on assessment of additional risk factors

*ACM* arrhythmogenic cardiomyopathy; *BrS* Brugada Syndrome; *CHD* congenital heart disease; *CPVT* catecholaminergic polymorphic ventricular tachycardia; *HCM* hypertrophic cardiomyopathy; *LGE* late gadolinium enhancement; *LVEF* left ventricular ejection fraction; *MRI* magnetic resonance imaging; *NIDCM* non-ischemic dilated cardiomyopathy); *SCA* sudden cardiac arrest; *SCD* sudden cardiac death; *VT* ventricular tachycardia

## Implantable cardioverter defibrillator in special populations

The ICD is a fundamental tool in the primary and secondary prevention of SCD in high-risk patients, and the previous and current evidence supports their use [26]. However, there are certain population groups that are usually underrepresented in clinical trials and therefore the evidence is not categorical or there is simply no evidence of its efficacy and the indication can generate debate [58]. Clarifying the criteria for its indication in these groups is necessary to improve the clinical performance of this therapy. In any case, we must have in mind that the information available on these “special groups” should always be interpreted with caution since the studies were not designed with the necessary statistical power to demonstrate their effect in subgroups. Having made these exceptions, we propose to make a brief review of the data available in some of these groups, with a fundamental focus on the use of ICD in primary prevention.

### Females

The efficacy of ICD in the female sex is a common cause of controversy mainly due to, and beyond other potential reasons, the fact that women are underrepresented in randomized clinical trials (8–29% of women included). In a systematic review of the evidence on the use of ICD in primary prevention of SCD published in 2014, 10 trials in which a subgroup analysis was possible were selected. In 9 of them, ICD was compared vs no ICD and in one CRT-D versus no ICD. All showed a decrease in overall mortality associated with ICD. However, in 7 of them in whom analysis by sex was done, this could not be demonstrated in women (HR 0.95 [95% CI 0.75–1.27]). The authors attribute this difference, in the first place, to the fact that less than 20% of patients included in these studies were female [54]. In addition, other investigators demonstrated a greater presence of comorbidities in the women included in clinical trials and a higher rate of hospital complications during implantation. For example, in HF, the existence of non-ischemic cardiomyopathy, a worse NYHA FC and a greater indication for CRT-D had a higher prevalence in women [59].

The MADIT-II study demonstrated that women were more likely to have advanced HF, high blood pressure, and diabetes mellitus [45]. By analyzing the data from 5 main clinical trials in primary prevention with ICD (MUSTT, MADIT-II, DEFINITE, SCD-HeFT, and COMPANION), other authors [60] showed that diuretic use, poorer functional class and non-white race were more frequent in included women. This meta-analysis demonstrated a lower mortality associated with the use of ICD in men, but not in women, although overall there were no differences. Another meta-analysis about the use of ICD in primary prevention, in patients with advanced HF, which included a total of 934 women, found no statistically significant differences in overall mortality in women who received ICD (HR 1.01 [CI 95% 0.76–1.33]). These authors refer to data from a Medicare cohort in the United States that included patients with HFrEF, where it was shown that only 8.6 / 1000 women vs 32.3 / 1000 men with an indication for ICD in primary prevention received it one year after HF was diagnosed [61].

In any case, it seems quite clear that women included in studies generally have a higher risk due to comorbidities and are also older, which determines a higher non-arrhythmic or non-cardiac mortality, which could be influencing the results [62]. These authors also review other potential reasons for this distinction in a recent publication where epidemiological differences (the incidence of SCD is 3 times higher in men) and clinical differences stand out: 2/3 of women with SCD have coronary heart disease versus 50% of men; men present more frequently with electrically shockable rhythms while women do so with electrical activity without pulse or asystole; the diagnosis of structural heart disease is less frequent in women, they are less frequently referred to a specialist and this option is less frequently offered to them, in addition to having a greater chance of being discharged from the hospital after an episode.

It has also been shown that in women with ICD, the incidence of appropriate shocks is significantly lower than in men [53]. In 3 classic trials on which the current indications for ICD in secondary prevention are based (AVID, CASH, CIDS), a detailed analysis by sex is not available [58].

In conclusion, although there may be sex differences in the response to this resource, probably in relation to reasons mentioned and others, the current guidelines do not make differences regarding sex in the indications for ICD implantation neither in primary nor secondary prevention [26]. It is very clear that it would be necessary to have studies with a better representation of the female sex.

### Elderly

In the same way that happens with women, elderly patients are usually underrepresented in clinical trials and the information available is linked to subgroup analysis with known limitations, therefore, their clinical performance is debatable in this population; the different age ranges used in the studies to consider a patient as “old” also contribute to this difficulty.

In a meta-analysis [54] mentioned above, including 6 clinical trials that differentiated between < 65 years and > 65 years, there were no statistically significant distinction in favor of the ICD in the older group (HR 0.93 [95% CI 0.73–1.20]). The results of another 6 trials that analyzed children and adults over 60 years, 70 years and 75 years were are also compared, and one trial that subdivided them between < 65 years, between 65 and 75, and > 75 years. All those trials were not analyzed all together, and separately, only 2 of them showed benefit in the older population (> 70 and > 75 years).

In MADIT II [45], a study about the evidence for the use of ICDs in primary prevention in IHD with a total of 1,232 randomized patients, 436 patients older than 70 years were included; the global results in favor of the ICD are well known (HR 0.69 [95% CI 0.51–0.93]), but also in the subgroup of older than 70 years the statistics were favorable.

In a cohort of 4,685 Medicare beneficiary patients in the USA, older than 65 years (mean age 75 years) with EF < 35%, who qualified for ICD implantation, 8% (376 patients) received the device at hospital discharge [63]; after 3 years of follow-up, the mortality of the ICD group was 38.1% while that of the control group was 52.3% (HR 0.71 [95% CI 0.56–0.91]). In a prospective analysis of 965 patients, ischemic and non-ischemic, with EF < 35%, 3-year mortality was compared between those who received an ICD (51%) and those who did not receive it [64], demonstrating a significant and consistent benefit with the use of the device in all age ranges (< 65, between 65 and 75, and > 75 years), and the benefit being slightly lower in the elderly population when there are comorbidities. These authors highlight the importance of considering comorbidities individually and not excluding patients exclusively based on age.

Another analysis that included more than 45,000 Medicare beneficiary patients (40% had a global average age > 75 years) and who were followed for 4 years [65], developed a nomogram based on 7 clinical parameters (age > 75 years, NYHA FC III, AF, DM, chronic obstructive pulmonary disease, CKD and LVEF) capable of identifying 10–20% of patients with high short-term mortality after implant. A simpler risk scale (*PACE risk score*) that considers 4 clinical conditions (presence of peripheral vascular disease [1 point], age > 70 years [1 point], creatinine > 2 mg / dl [2 point], or LVEF < 20% [1 point]) adequately predicted 1-year mortality. In the *PACE risk score*, a score > 3 multiplies mortality by 4 with respect to a score < 3 (16.5% vs 3.5%; *p* < 0.0001; C statistic: 0.795) [66].

In a report endorsed by different Scientific Societies from the USA [67] that sought to establish criteria for the appropriate use (when the benefit exceeds the potential risks) of the ICD, 3 categories of indications were defined according to a score developed based on the available evidence and the clinical criteria of a panel of experts against the different clinical situations: those where use is appropriate (score 7–9), where it may be appropriate (score 4–6) and those where it is rarely appropriate (score 1–3). In patients with LVEF < 30% and age between 80 and 89 years, they have a score of 4 (NYHA FC I) or 5 (NYHA FC II), while in > 90 years in NYHA FC I the score is 3 and in NYHA FC II and III it is 4. It is worth noting that this report takes into account the influence of age from 80 years of age. In the NCDR (National Cardiovascular Data Registry) [63, 68] corresponding to the years 2010 and 2011, it was found that 17% of the patients were older than 80 years and 0.9% were older than 90 years, a growing number compared to previous years and that perhaps has continued and continues to increase.

Although the incidence of SCD increases with age, so does non-sudden cardiovascular and noncardiac mortality potentially lowering effectiveness ICD use in this population [69]. The assessment of comorbidities and a life expectancy greater than 1 year are considerations repeatedly included in the current guidelines for the use of ICD, both for primary and secondary prevention [26].

In conclusion, age is another factor to consider, but it should not be an exclusive criterion. The realistic weighting of life expectancy, quality of life and comorbidities are more relevant in the group of elderly patients.

### Chronic kidney disease (CKD)

Patients with CKD have a higher incidence of SCD, but there are no data on the potential benefit that ICD could provide. The data come from observational studies and are conflicting; in addition, moderate-severe CKD and dialysis patients have been routinely excluded from randomized trials [26].

There are data, particularly in dialysis patients, showing that SCD is more frequently associated with bradycardia and asystole [70]. In any case, this comorbidity, especially when associated with others, limits the benefit that an ICD could provide. In the short-term mortality risk scale mentioned above (*PACE risk score*) (62), CKD defined as creatinine > 2 mg/dl is the only one of the 4 factors considered that is given a score of 2. A score > 3 multiplies by 4 the mortality compared to those with a value < 3 [66].

In another risk scale based on MADIT-II trial, long-term mortality (8 years) after device implantation is estimated and uses 5 risk factors: AF, NYHA FC > II, age > 70, QRS > 120 ms and urea > 0.56. This tool showed that those patients with > 3 factors did not benefit from the use of the ICD [71]. In addition, those patients with advanced CRF on dialysis have a higher risk of complications such as bleeding and infection related to the implant [72].

In the process for the preparation of the North American guidelines [27], an evidence review committee (Evidence Review Committee) was designated to analyze the issues in which there is no published systematic review, being relevant to a significant number of patients, where it is important to establish a recommendation on its risks-benefits, and possibly make a suggestion. This multidisciplinary committee conducted a meta-analysis of 5 observational studies, of which only 2 included patients with advanced CKD, and concluded that they suggest a relationship between ICD implantation and improved survival. Based on this, the use of ICDs in CRF on dialysis is not recommended [73]. In all other cases, the decision must be individualized, considering other comorbidities, functional status, preferences, etc. [26].

## Pediatrics

The use of ICD is a growing and important therapy in pediatric population with structural heart disease (congenital heart disease, non-ischemic cardiomyopathy) and channelopathies, although data about indications, outcomes, and complications is limited [74–76]. Recent studies have showed that in 50% of pediatric SCA survivors, the cause of event remains unknown despite an extensive evaluation [77]. Therefore, decision for implantation of an ICD is challenging, patient-specific factors and shared decision-making with parents are critically important [78]. The recent PACES expert consensus on the indications and management of cardiovascular implantable electronic devices in children [79], recommend ICD implantation based on specific cardiovascular disease, when reversible causes have been excluded and expected survival > 1 year. Main indications for ICD implantation for primary prevention in channelopathies, congenital heart disease and cardiomyopathies are summarized in Table 3.

Outcomes and complications after pediatric ICD implantation have been limited by the absolute number of patients in previous series. A retrospective review of the National Cardiovascular Data Registry ICD Registry addresses important information about patient, device characteristics and trends in ICD implantation from 2010 to 2016 in pediatric population [80]. According to data, there is an overall increase of ICD implantation for both primary and secondary prevention; non-ischemic cardiomyopathy is the most common cause for pediatric implantation (39%) followed by hypertrophic cardiomyopathy (17%) and long QT syndrome (13%). Most devices (60%) were implanted for primary prevention in patients without HF [76, 81]. The cause of this increasing trend is multifactorial, with more evidence of risk of SCD in pediatric populations and improved survival of patients with structural heart disease. Data about implantation in LA cohorts are scarce. The incidence of complications in children seems to be not significantly different than adult cohorts (2.3% vs 2.6%); however, pediatric patients are more likely to have cardiac arrest during or postprocedural (0.58% vs 0.25%, *p* = 0.004) than adult patients (76). Other small series have reported higher rates of pediatric in-hospital complications (10%-16.8%) [82]. Technical difficulties during ICD implantation are mainly related to patient characteristics such as low weight, Ebstein anomaly, single ventricle patients, and worse NYHA class. Other complications are inappropriate ICD shocks and early battery replacement [83].

## Implantable cardioverter defibrillator complications

The complications due to implantation of an ICD are estimated to be 3.0–9.5% [84]. They can be divided in two groups: Surgical and post-surgical complications.

The implant procedure is quite similar to a pacemaker implant and the principal difference is the size of the high voltage lead and the device. The lead is wider, rounded of defibrillation coils and the device is bigger and wider than a conventional PM, requiring a deeper subcutaneous pocket.

Most common surgical complications are bleeding, infections, pneumothorax, and heart (perforation) or vessel lesions. Infection is one of the feared complications, as the treatment is usually complicated, especially in devices implanted months or years before the complication. To avoid (when pocket is infected) or to cure the endocarditis, is mandatory to extract all the leads, and sometimes, they are attached to the veins or the heart. A Danish study evaluated the complications related to a cardiac implantable electronic device from May 2010 to April 2011. They followed 5.918 patients and found complications in 9.5% of the cases. High-risk factors for complications included the implant of a dual chamber CRT-D (RR 2.6), operators with low number of implants (RR 1.9), procedures out-of-hours or as an emergency (RR 1.5), underweight patients (RR 1.5), female gender (RR 1.3), low volume centers and the need of a device upgrade or lead revision (RR 1.3) [85].

The security of totally subcutaneous ICD was analyzed in the EFFORTLESS registry were they reported complications in 6.4% of patients, including erosion or extrusion of implanted electrode or pulse generator, hematoma, failure to convert spontaneous VF episode, inability to communicate with device, inappropriate shocks, oversensing, incision or superficial infection, pleural effusion, pneumothorax, premature battery depletion, shock delivered for non-VT/VF, system infection, suboptimal electrode/generator position and suture discomfort [86].

The replacements due to recalls are a risk factor for complications. A Canadian group collected ICD advisories (which are ICD malfunctions caused by failure of generator components, once made public) and reported abnormal battery depletion, short cuts, random memory error limiting delivery of therapies, memory chip alterations with impaired pacing and therapies or metal migration affecting pacing and therapies. The ICD advisories are reported between 0.009% and 2.6% of ICD implants. In 533 changed devices out 2.915 patients with recall devices, 8.1% had complications [87].

In 439 010 patients who underwent implantation of electronic cardiac devices (CRT) in USA between 2003 and 2013, it was reported at least one complication in 6.1% of cases. Predictors of complications included age > 65 and female gender (OR 1.19) while elective admission for implanting was a protective factor (OR 0.61) [88].

As the lead in an ICD is the most important source of complications, a real-world data was reviewed from an insurance database including 20.580 procedures (ICD or CRT-D implantation) performed between January 2003 and June 2015. They reported mechanical (2.165 [5.3%]) and infectious (771 [1.9%]) complications which were more likely in patients with a history of atrial fibrillation, DM, and renal disease. The risk of complications increased with subsequent device procedures. The authors found that 1 of 4 transvenous ICD leads had mechanical complications when followed up to 10 years [89].

## Recommendations for follow-up

As more and more people live longer with heart disease, ICD and CRT devices are implanted more frequently. It is important to make a proper follow-up of these devices, in order to ensure the correct functioning of the system and to obtain better clinical results.

For several decades, follow-up evaluation of implantable electronic cardiac devices has required an office evaluation for periodic device assessments. Actual technology has evolved to allow secure remote monitoring for almost all types of devices, and provides useful alerts in clinical practice, even if they are not as complete as an assessment of the devices in the office. For most patients, the majority of follow-up assessments of implantable devices can be done in person or remotely [90, 91]. After immediate post-implantation control, a first personal visit should take place between 4 weeks to 3 months after implantation. From then on, ideally one personal visit per year is recommended. Other follow-up assessments can be done in person or remotely (if available) [92].

Numerous studies, which have led to a meta-analysis, indicate that this remote monitoring can be as effective as clinical visits [93]. In the RM-ALONE study, participants were randomized to have their devices evaluated in the office or remotely every 6 months [94]. The results showed that, in an average follow-up of 21 months, there was no significant difference between the two groups in the main cardiac adverse events. Overall, a strategy to remotely monitor and interrogate devices appears to be just as safe and effective as a strategy that includes in-office visits twice a year. However, the cost of such devices is prohibitive for most patients.

The frequency of follow-up visits for patients with an implantable device will vary depending on the type of device, the implantation time and the patient's clinical condition. However, in general, most patients should make an annual follow-up visit in person, with one or more additional assessments (remote or in person) throughout the year. Patients who have received therapies (for example, ICD shock or anti tachycardia stimulation), as well as those whose devices are nearing the end of their battery life, may require more frequent monitoring.

ICD follow-up must be adapted to the clinical situation of every patient, especially based on the electrical stability. The follow-up can be obtained in person or remote and it must evaluate the clinical situation, skin wound and device integrity.

ICDs are usually implanted in patients at increased risk for ventricular arrhythmias or SCD, and because of that, ICD shocks are expected events during the long-term follow-up of these patients. Since a single ICD shock often represents the appropriate interruption of a sustained ventricular tachyarrhythmia, patients who receive an isolated ICD shock without loss of consciousness can be followed (in the office or remotely) within 24 to 48 h to ensure that the device is functioning properly. Other causes of ICD shock (such as supraventricular tachyarrhythmias or device malfunction) must be excluded and reassure the patient. A summary and recommendations are shown in Table 5. A Spanish version of this recommendations is presented in the Supplemental Material 1.

**Table 5:** Follow-up recommendations

Patient	Follow-up recommendation
Overall	Follow-up can be personal or remote (if available), according to the local protocol, but at least one assessment per year must be performed in the office
	The frequency of these may increase in certain clinical situations (for example, a device with a battery close to depletion or a suspected infection in the device)
Stable patients who have not received a shock from the ICD	Every six months. If remote, follow up in person at least once a year
Patients who receive a single shock	* without loss of consciousness and who feel well should be monitored in the office or remotely within 24 to 48 h
	if remote examination of the data reveals an appropriate shock and the patient is feeling well, a personal visit may not be necessary. However, if no follow-up in the office or remote is available for more than 24 to 48 h, the patient may need to be seen in the emergency room
Patients with electrical storms or with one single shock and is feeling poorly	Urgent evaluation in the emergency department should be indicated
Clinical situation is uncertain and / or if the patient is concerned or injured during the loss of consciousness	Patient should be seen at the clinic or in the emergency room
Patients with multiple shocks in a short period of time (minutes to hours) or have received a single shock and feels sick	Patient should be evaluated more urgently in the emergency department. All patients seen in the emergency room must undergo: - Anamnesis and physical examination - A 12-lead ECG - Additional laboratory tests according to the clinical picture

## What do the international clinical guidelines say about indications for implantable cardioverter defibrillator implantation for prevention of sudden death in heart failure?

Most international guidelines for implantation of an ICD take into account, along with clinical variables, the left ventricular ejection fraction that could easily be obtained in the LA population. Tables 6, 7, and 8 describe the main recommendations made from the guidelines from American, European, and Canadian Societies related to the treatment of HF [12, 27, 58, 95–97]. A Spanish version of this recommendations is presented in the Supplemental Material 1.

**Table 6:** American guidelines (*AHA/ACC 2013 -ACCF/AHA/HRS 2017-AHA/ACC/HFSA 2022*

Class of recommendation	Recommendation
CLASS I – LOE A	EF < 35%, at least 40 days post MI or 90 days post cardiac revascularization, NYHA FC II-III, under chronic OMT and with life expectancy > 1 year
	EF < 35%, non-ischemic cardiomyopathy, NYHA FC II–III with OMT and life expectancy > 1 year
CLASS I—LOE B	EF < 40%, NSVT due to previous MI and sustained VT or inducible VF in electrophysiological study, with life expectancy > 1 year
	EF < 30%, at least 40 days post infarction or 90 days post cardiac revascularization, NYHA FC I, in chronic OMT and with life expectancy > 1 year
CLASS IIa—LOE B	Outpatient in NYHA FC IV, candidate for transplantation or with a ventricular assist device in whom a survival > 1 year is reasonably predicted
	Non-ischemic cardiomyopathy due to Lamin A / C mutation with 2 or more risk factors (EF < 45%, NSVT, nonmissense mutation and male sex) with life expectancy > 1 year
CLASS IIb—LOE B	EF < 35%, non-ischemic cardiomyopathy, NYHA FC I, with OMT and life expectancy > 1 year
	Uncertain benefit in patients in whom survival is desired and are at high risk of non-sudden death, for example, due to frequent hospitalizations, comorbidities such as kidney failure or extreme frailty
CLASS III—LOE C	FC IV, refractory to medical treatment, not a candidate for transplantation, ventricular assist device or CRT

**Table 7:** European guidelines (ESC 2016–2021)

Class of recommendation	Recommendation
CLASS I – LOE A	EF < 35%, ischemic cardiomyopathy, NYHA FC II-III after > 3 months of OMT, at least 40 days post MI and with a life expectancy > 1 year
CLASS I—LOE A	EF < 35%, non-ischemic dilated cardiomyopathy, NYHA FC II-III after > 3 months of OMT, and with a life expectancy > 1 year
CLASS IIa—LOE A	Before replacing the device, the patient must be carefully reassessed by an experienced cardiologist because the clinical situation and the objectives of its management may have varied
CLASS IIb—LOE B	Consider a portable ICD in patients at high risk for MS for a limited period of time or while awaiting implantation
CLASS III—LOE A	Not recommended before 40 days of an acute MI
CLASS III—LOE C	Not recommended in patients in NYHA FC IV with severe symptoms refractory to pharmacological therapy unless they are candidates for CRT, ventricular assist device or transplant

**Table 8:** Canadian guidelines (Canadian Heart Falure Society)

Class of recommendation	Recommendation
Primary prevention	
Strong recommendation; high-quality evidence	We recommend consideration of primary ICD therapy in patients with: i. Ischemic cardiomyopathy, NYHA class II-III, EF 35%, measured at least 1 month post MI, and at least 3 months post coronary revascularization procedure ii. Ischemic cardiomyopathy, NYHA class I, and an EF 30% at least 1-month post MI, and at least 3 months post coronary revascularization procedure iii. Nonischemic cardiomyopathy, NYHA class II-III, EF 35%, measured at least 3 months after titration and optimization of GDMT
Strong recommendation; moderate-quality evidence	We recommend against ICD implantation in patients with NYHA class IV symptoms who are not expected to improve with any further therapy and who are not candidates for cardiac transplantation or mechanical circulatory support (MCS)
Secondary prevention	
Strong recommendation; high-quality evidence	We recommend an ICD be implanted in patients with HFrEF and a history of hemodynamically significant or sustained ventricular arrhythmia

## Main similarities and differences between American and European heart failure clinical guidelines in implantable cardioverter defibrillator indications

### Similarities

• EF < 35%, NYHA FC II-III, or NYHA FC II with EF < 30% in OMT [98]
• Secondary prevention [98]

### Differences

• Before changing the device, the patient must be carefully reassessed by an experienced cardiologist because the clinical situation and the objectives of its management may have varied [98]:
  - ACC / AHA: not mentioned
  - ESC: Class IIa
• Less than 40 days after an acute MI [98]:
  - ACC / AHA: not mentioned
  - ESC: class III

## Recommendations/conclusions

Numerous clinical studies have demonstrated the usefulness of ICD in both, primary and secondary prevention of SCD in HFrEF. There are currently precise indications and contraindications for the use of these devices.

In some LA countries, a low rate of implantation is correlated with low incomes, but this is not the case for all LA. Determinants of the low rates of ICD implantation in many LA countries is still a matter of research. VA remain one of the most common causes of cardiovascular death associated with HFrEF and different tools are available for stratifying the risk of SCD in this population. Many of these tools are readily available in LA (such as the 12-lead ECG, treadmill stress test, and Holter monitoring). As the number of Electrophysiologists in LA is not that high, LA Cardiologists play a very important role in the selection of cases and the proper referral to electrophysiologist.

## Supplementary Information

Below is the link to the electronic supplementary material.

Supplementary file1 (DOCX 30 KB)

## Abbreviations

ACC: American College of Cardiology
AF: Atrial fibrillation
AHA: American Heart Association
ARVC: Arrhythmogenic right ventricle cardiomyopathy
CAD: Coronary artery disease
CHD: Congenital heart disease
CIFACAH: Inter-American Council of Heart Failure and Pulmonary Hypertension
CKD: Chronic kidney disease
CPVT: Catecholaminergic polymorphic ventricular tachycardia
CRT-D: CRT/ICD
DCM: dilated cardiomyopathy
ECG: Electrocardiogram
EF: Ejection fraction
ElectroSIAC: Inter-American Council of Electrocardiography and Arrhythmias
ESC: European Society of Cardiology
FC: Functional class
HCM: Hypertrophic cardiomyopathy
HF: Heart failure
HFpEF: Heart failure with preserved ejection fraction
HFrEF: Heart failure with reduced ejection fraction
IASC: Inter-American Society of Cardiology
ICD: Implantable cardioverter-defibrillator
IHD: Ischemic heart disease
LA: Latin America
LAHRS: Latin American Heart Rhythm Society
LBB: Left bundle branch
LOE: Level of evidence
LQTS: Long QT syndrome
LV: Left ventricular
MI: Myocardial infarction
NSVT: Non-sustained ventricular tachycardia
NYHA: New York Heart Association
OMT: Optimal medical therapy
RR: Relative risk
SCD: Sudden cardiac death
SD: Sudden death
VA: Ventricular arrhythmias
VF: Ventricular fibrillation
VT: Ventricular tachycardia
