# Supplementary material for: Inter-American Society of Cardiology (CIFACAH-ELECTROSIAC) and Latin-American Heart Rhythm Society (LAHRS): multidisciplinary review on the appropriate use of implantable cardiodefibrillator in heart failure with reduced ejection fraction
Source: J Interv Card Electrophysiol. 2022 Dec 5;66(5):1211–29. doi: 10.1007/s10840-022-01425-4 (PMC10333140; doi:10.1007/s10840-022-01425-4)
Supplement: Supplementary file 1 — Supplementary file1 (DOCX 30 KB) [file 10840_2022_1425_MOESM1_ESM.docx]

**Supplemental material 1.** Spanish version of the recommendations given in the multidisciplinary review on the appropriate use of implantable cardiodefibrillator in heart failure with reduced ejection fraction by the Interamerican Society of Cardiology (CIFACAH - ELECTROSIAC) and Latin-American Heart Rhythm Society (LAHRS)

Tabla 1. Recomendaciones para implantar un DCI

| **Clase de recomendación** | **Recomendación** |
| --- | --- |
| **PREVENCIÓN PRIMARIA** | |
| IA | Se recomienda un DCI para reducir el riesgo de muerte súbita y mortalidad por todas las causas en pacientes con insuficiencia cardiaca (IC) sintomática (pacientes de la clase II -III de la *NYHA*) de etiología isquémica (a menos que hayan tenido un IM en los 40 días anteriores) (ver más adelante) y una de fracción de eyección del ventrículo izquierdo (FEVI) ≤35%; esto, a pesar de proporcionar ≥3 meses de tratamiento médico óptimo (TMO); se espera que sobrevivan, sustancialmente, más de un año con un buen estado funcional. |
| IIa A | Debe considerarse la posibilidad de utilizar un DCI para reducir el riesgo de muerte súbita y mortalidad por todas las causas en pacientes con insuficiencia cardiaca sintomática (clase II-III de la *NYHA*) de etiología no isquémica y de una FEVI ≤35%. A pesar de proporcionar ≤3 meses de TMO, se espera que sobrevivan sustancialmente más de un año con un buen estado funcional. |
| IIa A | Un cardiólogo experimentado debe evaluar cuidadosamente a los pacientes antes del reemplazo del generador porque pueden haber cambiado los objetivos de manejo, las necesidades del paciente y el estado clínico. |
| IIb B | Se puede considerar el uso de un DCI portátil en pacientes con: a) IC que corren el riesgo de sufrir una muerte súbita cardíaca durante un período limitado o b) como un puente hacia la implantación de un dispositivo. |
| III A | No se recomienda la implantación de DCI dentro de los 40 días siguientes a un IM. Esto se debe a que, la implantación en ese momento no mejora el pronóstico. |
| III C | El tratamiento con DCI no se recomienda en pacientes de la clase IV de la *NYHA* con síntomas graves, refractarios al tratamiento farmacológico; a menos de que sean candidatos para recibir un tratamiento de resincronización cardíaca (TRC), para recibir un dispositivo de asistencia ventricular (DAV) o para recibir un trasplante cardíaco. |
| **PREVENCIÓN SECUNDARIA** | |
| IA | Se recomienda un Desfibrilador cardioversor implantable (DCI) para reducir el riesgo de muerte súbita y mortalidad por todas las causas en pacientes que se han recuperado de una arritmia ventricular —causante de una inestabilidad hemodinámica— y que se espera que sobrevivan durante >1 año con un buen estado funcional; en ausencia de causas reversibles o a menos que la arritmia ventricular haya ocurrido <48 h después de un infarto de miocardio (IM). |

Tabla 2. Indicaciones de implante de DCI en prevención primaria durante la edad pediátrica

|  | **Recomendaciones** | |
| --- | --- | --- |
| **Recomendación general** | **IIb** | Pacientes con enfermedades cardiovasculares genéticas y factores de riesgo de paro cardíaco súbito (PCS) o con mutaciones patogénicas y antecedentes familiares de PCS recurrente. |
| **SQTL** | **IIb** | Pacientes con factores de riesgo clínicos establecidos y/o mutaciones patogénicas. |
| **TVPC** | **IIb** | Pacientes con taquicardia ventricular (TV) polimórfica/bidireccional; a pesar del tratamiento farmacológica óptimo con o sin denervación simpática cardíaca (DCS) |
| **SBr** | **IIa** | Pacientes con un patrón ECG Brugada tipo I espontáneo y síncope reciente presuntamente debido a arritmias ventriculares. |
|  | **IIb** | Pacientes con síncope posiblemente debido a arritmias ventriculares y que, con fármacos usados en pruebas de provocación, presenta un ECG con patrón de Brugada tipo I. |
| **MCH** | **Iia** | Niños con >1 factor de riesgo primario, síncope, hipertrofia ventricular izquierda masiva, TV no sostenida o antecedentes familiares de MCH temprana relacionada con MCS; tras considerar las posibles complicaciones de la colocación de DCI a largo plazo. |
|  | **IIb** | Sin los factores de riesgo mencionados, pero con factores de riesgo secundarios para PCS como: una extensa RTG en resonancia magnética cardíaca (RMC) o una disfunción sistólica. |
| **MCDNI** | **IIb** | Síncope o una FEVI < 35% a pesar de la terapia médica óptima (TMO). |
| **ECC** | **IIb** | Síncope inexplicable en presencia de una disfunción ventricular, una TV no sostenida o arritmias inducibles en el estudio electrofisiológico. |
|  | **IIb** | Los pacientes con FEVI única o sistémica < del 35%, especialmente, en presencia de factores de riesgo adicionales como TV, síncope arrítmico o insuficiencia valvular auriculoventricular (AV) sistémica grave. |
| **MCA** | **IIb** | La MCA hereditaria se asocia a un mayor riesgo de MSC según la evaluación de factores de riesgo adicionales. |

*AV (Auriculoventricular); CTG (Captación tardía de gadolinio); DCS (Denervación simpática cardíaca); ECC (Enfermedad cardíaca congénita); FEVI (Fracción de eyección del ventrículo izquierdo); MCA (Miocardiopatía arritmogénica); MCDNI (Miocardiopatía dilatada no isquémica); MCH (Miocardiopatía hipertrófica); MSC (Muerte súbita cardíaca); RMC (Resonancia magnética cardíaca);RTG (Retención tardía de compuestos de gadolinio); SBr (Síndrome de Brugada); SCA (Paro cardíaco repentino); SQTL (Síndrome del QT Largo); TMO (Terapia médica óptima); TVPC (Taquicardia Ventricular Polimórfica Catecolaminérgica)*

Tabla 3. Recomendaciones de seguimiento

| **Paciente** | **Recomendación de seguimiento** |
| --- | --- |
| En general | De acuerdo con el protocolo local, el seguimiento puede ser personal o remoto (si está disponible). Sin embargo, al menos una de las evaluaciones anuales debe realizarse en una consulta presencial. |
|  | La frecuencia de estos chequeos médicos puede aumentar en determinadas situaciones clínicas. Por ejemplo, un dispositivo con la batería a punto de agotarse o la sospecha de una infección asociada al dispositivo. |
| Pacientes estables que no han recibido una descarga eléctrica o choque del DCI | Un examen médico de control cada seis meses.  Si es a distancia, hacer un seguimiento en persona al menos una vez al año. |
| Pacientes que reciben un solo choque | *Si el paciente no tiene pérdida de conciencia y afirma que se siente bien, debe ser monitoreado en la oficina o de forma remota dentro de las siguientes 24 a 48 horas. |
|  | Si la evaluación remota de los datos revela un choque adecuado y el paciente se siente bien, puede no ser necesaria una visita personal. Sin embargo, si no hay disponibilidad de seguimiento en consulta personal o a distancia en un plazo de 24 a 48 horas o más, puede ser necesario atender al paciente en urgencias. |
| Los pacientes con tormentas eléctricas o que con una sola descarga se sienten mal. | Debe indicarse una evaluación inminente en el servicio de urgencias. |
| La situación clínica es incierta, y si el paciente está preocupado o se lesionó durante la pérdida del conocimiento. | El paciente debe ser visto en el consultorio o en la sala de emergencias. |
| Los pacientes con múltiples choques en poco tiempo (de minutos a horas) o que han recibido un solo choque y se sienten enfermos. | Los pacientes deben ser evaluados con mayor prioridad en el servicio de urgencias. Todos los pacientes atendidos en la sala de emergencias deberán ser sometidos a:   - Anamnesis y examen físico - Un ECG de 12 derivaciones - Pruebas de laboratorio adicionales según el cuadro clínico. |

Tabla 4. Directrices estadounidenses (*AHA/ACC 2013 -ACCF/AHA/HRS 2017-AHA/ACC/HFSA 2022*

| **Clase (intensidad) de recomendación** | **Recomendación** |
| --- | --- |
| CLASE I – LOE A | Fracción de eyección (FE) <35%, al menos 40 días después del IM o 90 días después de una revascularización cardíaca, clase funcional (CF) II-III de la NYHA, bajo TMO habitual y con una esperanza de vida> 1 año. |
|  | FE <35%, miocardiopatía no isquémica, CF II-III de la NYHA, con TMO y esperanza de vida > 1 año. |
| CLASE I - LOE B | FE <40%, taquicardia ventricular no sostenida (TVNS) por IM previo y TV sostenida o FV inducible en estudio electrofisiológico, con esperanza de vida> 1 año. |
|  | FE <30%, al menos 40 días después del infarto o 90 días después de la revascularización cardíaca, CF I de la NYHA, en TMO frecuente y con esperanza de vida> 1 año. |
| CLASE IIa - LOE B | Paciente ambulatorio en CF IV de la NYHA, candidato a trasplante o con un dispositivo de asistencia ventricular en el que se predice razonablemente una supervivencia> 1 año. |
|  | Miocardiopatía no isquémica debida a la mutación de las láminas A/C con dos o más factores de riesgo (FE <45%, TVNS, mutación sin sentido y sexo masculino) con esperanza de vida> 1 año. |
| CLASE IIb - LOE B | FE <35%, miocardiopatía no isquémica, CF I de la NYHA, TMO y esperanza de vida > 1 año. |
|  | Beneficio incierto en pacientes en los que se desea la supervivencia y tienen un alto riesgo de muerte no súbita, por ejemplo, debido a hospitalizaciones frecuentes, comorbilidades como la insuficiencia renal o la fragilidad extrema. |
| CLASE III - LOE C | FC IV de la NYHA, refractaria al tratamiento médico, no candidato para trasplante, dispositivo de asistencia ventricular o TRC |

Tabla 5. Directrices europeas (CES 2016-2021)

| **Clase de recomendación** | **Recomendación** |
| --- | --- |
| CLASE I – LOE A | FE <35%, miocardiopatía isquémica, CF II-III de la NYHA después de > 3 meses de TMO, al menos 40 días después del IM y con una esperanza de vida > 1 año. |
| CLASE I - LOE A | FE <35%, miocardiopatía dilatada no isquémica, CF II-III de la NYHA después de > de 3 meses de TMO, y con una esperanza de vida > 1 año. |
| CLASE IIa - LOE A | Antes de sustituir el dispositivo, un cardiólogo experimentado debe reevaluar cuidadosamente al paciente porque pueden haber variado tanto la situación clínica como los objetivos de manejo del dispositivo. |
| CLASE IIb - LOE B | Se debe considerar un DCI portátil en pacientes con alto riesgo de muerte súbita durante un período de tiempo limitado o mientras esperan la implantación. |
| CLASE III - LOE A | No se recomienda antes de 40 días posteriores a un infarto agudo de miocardio (IM) |
| CLASE III - LOE C | No se recomienda en pacientes en CF IV de la NYHA con síntomas graves refractarios al tratamiento farmacológico; a menos que sean candidatos para TRC, para un dispositivo de asistencia ventricular o para trasplante. |

Tabla 6. Directrices canadienses (Sociedad Canadiense de Cardiología)

| **Clase de recomendación** | **Recomendación** |
| --- | --- |
| **PREVENCIÓN PRIMARIA** | |
| Fuerte recomendación; Evidencia de alta calidad | Recomendamos que se considere la terapia primaria con DCI en pacientes con:  i. Miocardiopatía isquémica, clase II-III de la NYHA, FE del 35% —medida al menos un mes después del IM— y, al menos, tres meses después del procedimiento de revascularización coronaria  ii. Miocardiopatía isquémica, clase I de la NYHA, y una FE al 30% —al menos 1 mes después del IM— y, al menos, tres meses después del procedimiento de revascularización coronaria.  iii. Miocardiopatía no isquémica, clase II-III de la NYHA, con un FE del 35% — medida al menos tres meses después de la titulación de fármacos— y optimización de terapia médica dirigida por las guías (TMDG). |
| Fuerte recomendación; Evidencia de calidad moderada | Se desaconseja la implantación de un DCI en pacientes con síntomas de clase IV de la NYHA que no se espera que mejoren con ningún otro tratamiento adicional y que no son candidatos a un trasplante cardíaco o a un soporte circulatorio mecánico (SCM). |
| **PREVENCIÓN SECUNDARIA** | |
| Fuerte recomendación; Evidencia de alta calidad | Recomendamos la implantación de un DCI en pacientes con Fracción de eyección reducida (IC-FER) y antecedentes de arritmia ventricular hemodinámica significativa o sostenida. |
